# 11βHSD1 Inhibition with AZD4017 Improves Lipid Profiles and Lean Muscle Mass in Idiopathic Intracranial Hypertension

**DOI:** 10.1210/clinem/dgaa766

**Published:** 2020-10-24

**Authors:** Rowan S Hardy, Hannah Botfield, Keira Markey, James L Mitchell, Zerin Alimajstorovic, Connar S J Westgate, Michael Sagmeister, Rebecca J Fairclough, Ryan S Ottridge, Andreas Yiangou, Karl-Heinz H Storbeck, Angela E Taylor, Lorna C Gilligan, Wiebke Arlt, Paul M Stewart, Jeremy W Tomlinson, Susan P Mollan, Gareth G Lavery, Alexandra J Sinclair

**Affiliations:** 1 Institute of Metabolism and Systems Research, College of Medical and Dental Sciences, University of Birmingham, Birmingham, UK; 2 Institute of Inflammation and Ageing, College of Medical and Dental Sciences, University of Birmingham, Birmingham, UK; 3 Institute of Clinical Sciences, College of Medical and Dental Sciences, University of Birmingham, UK; 4 Centre for Endocrinology, Diabetes and Metabolism, Birmingham Health Partners, Birmingham, UK; 5 Department of Neurology, University Hospitals Birmingham NHS Foundation Trust, Queen Elizabeth Hospital, Birmingham, UK; 6 Emerging Innovations Unit, Discovery Sciences. BioPharmaceuticals R&D, AstraZeneca, Cambridge, UK; 7 Birmingham Clinical Trials Unit, Institute of Applied Health Research, College of Medical and Dental Sciences, University of Birmingham, Birmingham, UK; 8 Department of Biochemistry, Stellenbosch University, Stellenbosch, Matieland, South Africa; 9 NIHR Birmingham Biomedical Research Centre, University of Birmingham and University Hospitals Birmingham NHS Foundation Trust, Birmingham, UK; 10 Medical School, University of Leeds, Leeds, UK; 11 Oxford Centre for Diabetes, Endocrinology & Metabolism (OCDEM), NIHR Oxford Biomedical Research Centre, University of Oxford, Churchill Hospital, Oxford, UK

**Keywords:** 11b-HSD1, AZD4017, 11b-HSD1 inhibitor, Idiopathic intracranial hypertension, cortisol

## Abstract

**Background:**

The enzyme 11β-hydroxysteroid dehydrogenase type 1 (11β-HSD1) determines prereceptor metabolism and activation of glucocorticoids within peripheral tissues. Its dysregulation has been implicated in a wide array of metabolic diseases, leading to the development of selective 11β-HSD1 inhibitors. We examined the impact of the reversible competitive 11β-HSD1 inhibitor, AZD4017, on the metabolic profile in an overweight female cohort with idiopathic intracranial hypertension (IIH).

**Methods:**

We conducted a UK multicenter phase II randomized, double-blind, placebo-controlled trial of 12-week treatment with AZD4017. Serum markers of glucose homeostasis, lipid metabolism, renal and hepatic function, inflammation and androgen profiles were determined and examined in relation to changes in fat and lean mass by dual-energy X-ray absorptiometry.

**Results:**

Patients receiving AZD4017 showed significant improvements in lipid profiles (decreased cholesterol, increased high-density lipoprotein [HDL] and cholesterol/HDL ratio), markers of hepatic function (decreased alkaline phosphatase and gamma-glutamyl transferase), and increased lean muscle mass (1.8%, *P* < .001). No changes in body mass index, fat mass, and markers of glucose metabolism or inflammation were observed. Patients receiving AZD4017 demonstrated increased levels of circulating androgens, positively correlated with changes in total lean muscle mass.

**Conclusions:**

These beneficial metabolic changes represent a reduction in risk factors associated with raised intracranial pressure and represent further beneficial therapeutic outcomes of 11β-HSD1 inhibition by AZD4017 in this overweight IIH cohort. In particular, beneficial changes in lean muscle mass associated with AZD4017 may reflect new applications for this nature of inhibitor in the management of conditions such as sarcopenia.

Glucocorticoids are a class of pleiotropic steroid hormones with diverse effects on systemic metabolism that in excess promote insulin resistance, obesity, hypertension, and dyslipidemia (1). The enzyme 11β-hydroxysteroid dehydrogenase type 1 (11β-HSD1) primarily converts the inactive glucocorticoid cortisone to its active counterpart cortisol (2). Its dysregulation and elevated activity within peripheral tissues results in an increased exposure to active glucocorticoids, and have been implicated in a wide array of diseases including type 2 diabetes mellitus, cardiovascular disease, metabolic syndrome, osteoporosis, hypertension, nonalcoholic fatty liver disease, and Alzheimer’s disease (3-6).

Consequently, multiple pharmacological 11β-HSD1 inhibitors, including MK-0736, MK-0916, RO-151, ABT-384, and INCB13739 have been developed for the management of these conditions, and examined in phase II clinical trials (7-13). Several of these studies demonstrated that these drugs are well tolerated and yield improvements in measures of glycated hemoglobin (HbA1c), low-density lipoprotein (LDL) cholesterol, high-density lipoprotein (HDL) cholesterol, liver fat content, and body weights when administered for up to 12 weeks, (7, 10, 12, 13). While these studies demonstrated differences in the efficacy of this class of inhibitors between different patient cohorts, including type 2 diabetes and hypertension, the magnitude of this benefit proved insufficient compared with existing standard drugs for disease management. However, the diverse metabolic targets of 11β-HSD1 inhibitors remain a notable and unique strength in their application. Further refinements to 11β-HSD1 inhibitors have continued to identify new compounds with more favorable therapeutic properties. The nicotinic amide–derived carboxylic acid class of 11β-HSD1 inhibitors (including AZD4017) are 1 such family of compounds that show favorable potency, selectivity, and pharmacokinetic properties (median inhibitory concentration [IC_50_] 0.007 µM) (14).

One particular area of interest identified for 11β-HSD1 inhibitors has been the management of idiopathic intracranial hypertension (IIH). This condition, characterized by raised intracranial pressure, papilledema, chronic headaches, and reduced quality of life also shows significant correlation between changes in 11β-HSD1 activity in the choroid plexus and changes in intracranial pressure, indicating a possible role in disease pathophysiology (15-17). In IIH cohorts, this occurs in combination with metabolic dysregulation and obesity, which are themselves risk factors for developing IIH and potential targets for 11β-HSD1 inhibitors (18, 19). The application of AZD4017 in the management of IIH and raised intracranial pressure has now been examined in a phase II clinical trial (6, 20). Here, AZD4017 was well tolerated and demonstrated a relationship between 11β-HSD1 inhibition and reduction in intracranial pressure. However, the wider metabolic and inflammatory effects of AZD4017 in this IIH patient cohort have yet to be fully examined.

This study investigates the consequences of 12-week 11β-HSD1 inhibition on insulin sensitivity, body weight and composition, lipid profiles, liver and renal function, systemic and central nervous system inflammation, and glucocorticoid and androgen pathways in a cohort of young obese women with a diagnosis of IIH.

## Patients and Methods

### Study conduct

This multicenter UK study was conducted from March 2014 to December 2016. The National Research Ethics Committee York and Humber-Leeds West gave ethical approval (13/YH/0366). All patients provided written informed consent in accordance with the declaration of Helsinki. The trial is registered at Clinicaltrials.gov NCT02017444; European Clinical Trials Database (EudraCT Number: 2013-003643-31). The detailed clinical trial methodology has been published (20).

### Study population

Obese women aged 18 to 55 years were recruited who were otherwise healthy except for a diagnosis of IIH (21, 22). Exclusion criteria were a significant past medical history including thyroid disease, other complex endocrine disease, renal failure, liver failure, hypertension (blood pressure >160 mmHg systolic), systemic (including vaginal/rectal) glucocorticoid treatment, hormone-based medication, including hormone contraceptives, probenecid intake, and any medical/surgical procedure or trauma within 4 weeks prior to study enrolment. Coexisting medications are reported elsewhere (all supplementary material and figures are located in a digital research materials repository (23)).

### Study design

A UK multicenter double-blind placebo-controlled randomized controlled trial with a 12-week dosing duration and 4-week follow-up off drug (total duration 16 weeks). Participants were randomized to either an oral selective 11β-HSD1 inhibitor, AZD4017, at 400 mg twice daily, or a matched placebo for 12 weeks.

#### Randomization:

The IIHDT randomization notepad was used, and after venesection on the baseline day the number was allocated by University Hospitals Birmingham pharmacy (20). Patients were allocated a trial number randomly by phone, using block-of-6 randomization. The study investigators, nurses, and participants were all blinded to the treatment allocation during the trial.

### Assessments

All participants underwent a detailed medical history and examination. Assessments were conducted at 10 visits over a 16-week period (full assessment protocol detailed in (6 and 20)). All blood samples were collected following an overnight fast (from midnight). Lumbar punctures were conducted in the left lateral decubitus with knees bent at a 90° angle or more and intracranial pressure recorded before cerebrospinal fluid (CSF) was collected (up to 15 mL). Body mass index (BMI) was measured from height (Seca Leicester height measure, Birmingham, UK) and weight (Seca 677 electronic wheelchair scale, Birmingham, UK) using the following formula: BMI = (weight (kg)/height (m)^2^). Adverse events were recorded along with drug compliance (unused medication documented).

### Fasting insulin and HOMA2-IR

Fasting insulin (Mercodia, Uppsala, Sweden) was measured using a commercially available assay, according to the manufacturer’s instructions. Homeostasis model assessment of insulin resistance (HOMA2-IR) was calculated using the program HOMA calculator v2.2.3 (24).

### Blood and cerebrospinal fluid biochemical analysis

The following fasted bloods were processed at the hospital laboratory: glucose, HbA1c, cholesterol, HDLs, and triglycerides, liver and kidney function tests including, alkaline phosphatase (ALP), gamma-glutamyl transferase (GGT), aspartate transaminase (AST), alanine aminotransferase (ALT), bilirubin, creatinine, estimated glomerular filtration rate (eGFR), and creatinine kinase (CK). Samples not analyzed immediately were centrifuged (10 minutes at 1500*g* at 4°C) aliquoted and stored at –80°C. CSF samples were centrifuged (800*g* for 10 minutes at 4°C) and the supernatant was aliquoted and stored at –80°C. All samples processed only underwent a single freeze–thaw cycle.

### Cytokines, adipokines, and bone marker analysis

Serum (1 in 2 dilution) and CSF interleukin (IL)-8, leptin, and MCP-1 were measured using the Procarta Multiplex immunoassay (Thermo Fisher Scientific) with lower limits of detection of 1.2 pg/mL, 2.0 pg/mL and 0.6 pg/mL respectively. Serum (1 in 2 dilution) and CSF IL-1β, IL-10, IL-6, and TNF-α were measured using the Procarta High Sensitivity Multiplex immunoassay (Thermo Fisher Scientific) with lower limits of detection of 0.145 pg/mL, 0.039 pg/mL, 0.038 pg/mL, 0.215 pg/mL. respectively. Serum (1 in 500 dilution) and CSF adiponectin was measured using the Procarta Singleplex immunoassay (Thermo Fisher Scientific) with lower limits of detection of 4.6 pg/mL. Serum osteocalcin (OC) and sclerostin (SC) were measured using the Milliplex MAP Human Bone Magnetic Bead Panel (Millipore) with lower limits of detection of 68.5 pg/mL and 31.1 pg/mL, respectively. All assays were conducted according to the manufacturer’s instructions and were analyzed using a Luminex 100/200 system.

### Serum steroid profiling by liquid chromatography-tandem mass spectrometry

Serum steroids were analyzed using liquid chromatography-tandem mass spectrometry (LC-MS/MS), as previously described (25-27). An internal standard mixture was added to 400 µL of serum. Steroids were extracted via liquid/liquid extraction with 2 mL of tert-butyl methyl ether (MTBE). The MTBE layer was removed, evaporated to dryness, and reconstituted in methanol/water prior to LC-MS/MS analysis. The extracts were analyzed on a Xevo TQ-S triple quadrupole mass spectrometer (Waters) coupled to an Acquity ultrahigh-performance liquid chromatography system (UPLC) (Waters). Steroids were separated on a HSS T3 (1.8 µm) column (Waters) using a methanol/water gradient (both with 0.1% formic acid). Starting conditions was 45% methanol, which was held for 1 minute, followed by a linear gradient to 75% methanol at 5 minutes. Subsequently, the column was washed at 98% methanol and reconditioned at starting condition prior to the next injection. Steroids were identified and quantified via comparison to reference standards; positive identification was confirmed via matching retention time and two identical mass transitions. Steroids quantified were testosterone, androstenedione, dehydroepiandrosterone (DHEA), cortisol, 11-ketoandrostenedione and 11β-hydroxyandrostenedione. The calibration series ranged from 0.01 to 250 ng/mL (including a blank and a 0 ng/mL calibrator).

### Dual-energy X-ray absorptiometry

Dual-energy X-ray absorptiometry (DXA) was performed using a total-body scanner (QDR 4500; Hologic), as previously described (28). Patients with metal prosthetics or implants were included, and tissue overlying the prosthesis was excluded from analysis. Scans were checked for accuracy of fields of measurement. Regional fat mass was analyzed as described previously (28). The precision of total fat mass measures in terms of coefficient of variation (CV) was less than 3%, and for regional fat analyses it was less than 5%. Regional fat data were expressed as a percentage of the total body fat.

### Statistical analysis

The trial was powered to assess clinical efficacy in IIH but not changes in metabolic parameters (20). All patients were invited for all assessments within the trial. Any absent patient data reflected individual choice to omit a test. Statistical analysis was performed using Graphpad prism 8 (Graphpad Software Inc). The data are presented as mean and standard error of the mean. Differences between values at baseline and 3 months were tested using 2-way analysis of variance or mixed effects analysis to account for missing data points and 95% confidence intervals noted. Multiple comparisons were conducted to compare week 0 with week 12 in each of treatment groups, and *P* values were adjusted using Sidak’s multiple comparison test. Spearman’s rank correlation coefficient was used for assessing correlations. Results were judged statistically significant at *P* < .05.

## Results

### Patient cohort and clinical characteristics

**Figure 1.**
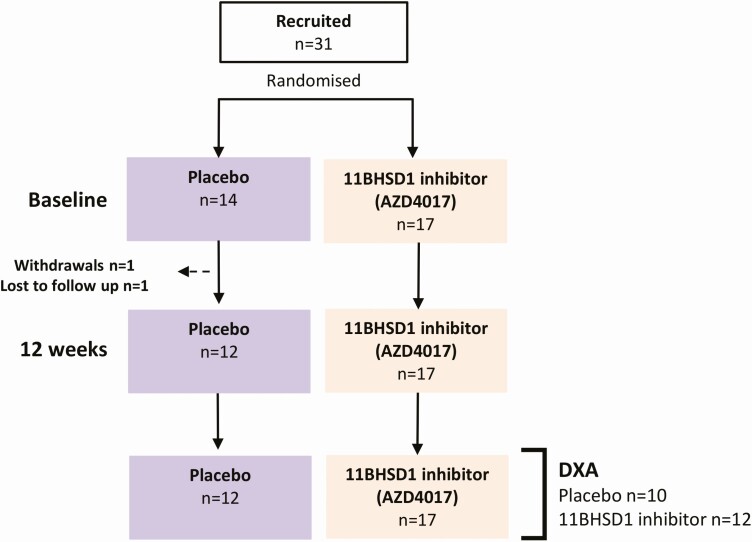
Trial design and participant numbers for double-blind, placebo-controlled, randomized controlled trial in IIH using the selective 11β-HSD1 inhibitor AZD4017.

This study was a double-blind randomized placebo-controlled trial of the 11β-HSD1 inhibitor AZD4017 for 12 weeks in 31 women with active IIH. Seventeen participants received AZD4017 (no withdrawals) and 14 participants received placebo (1 withdrawal due to an adverse event deemed unrelated to the study, 1 loss to follow-up) (Fig. 1). Complete study design, pharmacodynamic efficacy of 11β-HSD1 inhibition, effect on IIH disease measures, and adverse event profile have previously been reported (6). In brief, the patient cohort had an average age of 31.2 ± 6.9 years, weight of 102.6 ± 32.3 kg, and BMI of 39.2 ± 12.6 kg/m^2^. There were no significant differences in baseline characteristics between the trial arms (Table 1).

**Table 1.** Baseline characteristics of idiopathic intracranial hypertension (IIH) cohort receiving either placebo or the 11β-HSD1 inhibitor AZD4017

	Placebo (n = 14)	AZD4017 (n = 17)	Total (n = 31)
Age in years (SD)	32.4 (8.0)	30.1 (5.9)	31.2 (6.9)
Ethnicity, n (%)			
White British	13 (93)	16 (94)	29 (94)
Asian/Asian British – Pakistani	0 (0)	1 (6)	1 (3)
Asian/Asian British – 0ther Asian	1 (7)	0 (0)	1 (3)
Weight, kg (SD)	108.4 (42.3)	97.9 (21.3)	102.6 (32.3)
BMI (weight (kg)/ height (m^2^) (SD)	41.2 (16.6)	37.3 (7.2)	39.2 (12.6)

No significant differences were found between groups for any of the parameters.

Abbreviations: BMI, body mass index; SD, standard deviation.

### Pharmacological efficacy of AZD4017

The pharmacological properties of AZD4017 to inhibit 11β-HSD1 with high efficacy and specificity have previously been validated in phase I and II trials (6, 14). In brief, AZD4017 inhibits human 11β-HSD1 at IC_50_ = 0.007 μM, contrasting with IC_50_ > 30 μM for closely related enzymes 11β-HSD2, 17β-HSD1, or 17β-HSD3 (14). Treatment with AZD4017 in the clinical trial resulted in 70% suppression of systemic 11β-HSD1 activity as measured by 24-hour urinary 5α-tetrahydrocortisol + tetrahydrocortisol):tetrahydrocortisone ratio (0.27 ± 0.29 vs 0.90 ± 0.28; *P* < .0001) (6). Liver is the main site for 11β-HSD1 activity in vivo and efficacy for this tissue was assessed separately through a non-invasive pharmacodynamic study. Hepatic 11β-HSD1 activity was suppressed by 85.9% as measured by the conversion rate of orally administered prednisone to prednisolone (area under the curve analysis 228 ± 99 vs 1738 ± 142; *P* < .0001) (6). Clinical endpoint data for AZD4017 have been reported from 1 phase II trial. Here, in a patient cohort with idiopathic intracranial hypertension receiving 400 mg of oral AZD4017 twice daily, versus placebo over 12 weeks, the primary outcome was lumbar puncture opening pressure. Reduction of serum cortisol:cortisone ratio observed with AZD4017 treatment correlated with decreased intracranial pressure (r = 0.70; *P* = .005; n = 15) (6). Intracranial pressure reduced in those taking AZD4017 (mean change: −4.3 cmH_2_O [SD = 5.7], *P* = .009) but there was no significant difference between arms (–2.8 cmH_2_O; 95% confidence interval: –7.1 to 1.5 cmH_2_O; *P* = .2; n = 31) (6). In summary, the data demonstrate that AZD4017 specifically inhibits 11β-HSD1 activity in vivo.

### Glucose homeostasis and Lipid metabolism

**Figure 2.**
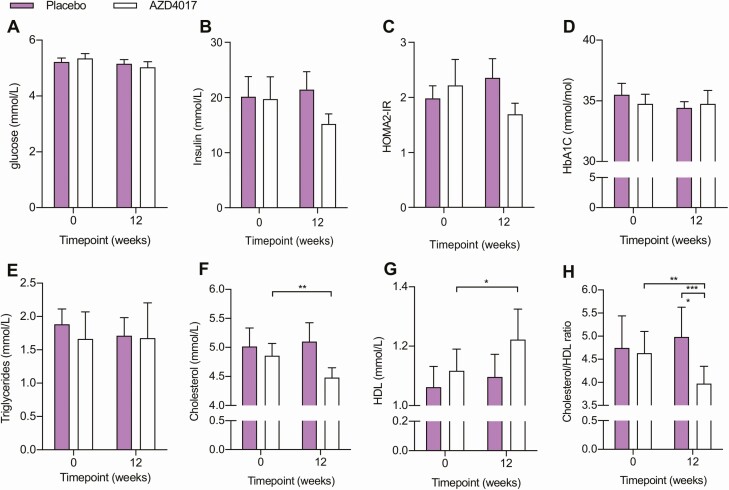
Metabolic changes following 12 weeks of AZD4017 treatment in IIH patients. Histograms showing glucose (A), insulin (B), HOMA2-IR (C), HbA1c (D), triglycerides (E), cholesterol (F), HDL (G), and the cholesterol/HDL ratio (H) in IIH patients before and after 12 weeks of either placebo or AZD4017 treatment. Data presented as mean ± SD. *P < .05, **P < .01, ***P < .001.

We examined serum measures of both glucose homeostasis and lipid metabolism (Fig. 2A-H). No significant changes in fasting glucose, fasting insulin, HOMA2-IR and HbA1c were apparent in either treatment group at 12 weeks relative to baseline (Fig. 2A-D and (23). Analysis of lipid metabolism revealed reduced cholesterol (–7.9%, *P* < .01), increased HDL (8.2%1, *P* < .05) and a decreased cholesterol/HDL ratio (–17.4%, *P* < .01) at 12 weeks relative to baseline in the AZD4017 group, but not in the placebo group (Fig. 2E-H and (23)). Triglyceride levels remained unchanged in both groups. Together, these data indicate that while glucose homeostasis appeared to be largely unaffected in this normoglycemic cohort, 12 weeks of AZD4017 treatment had beneficial effects on serum cholesterol profiles.

### Liver and renal function and systemic and central nervous system inflammation

**Figure 3.**
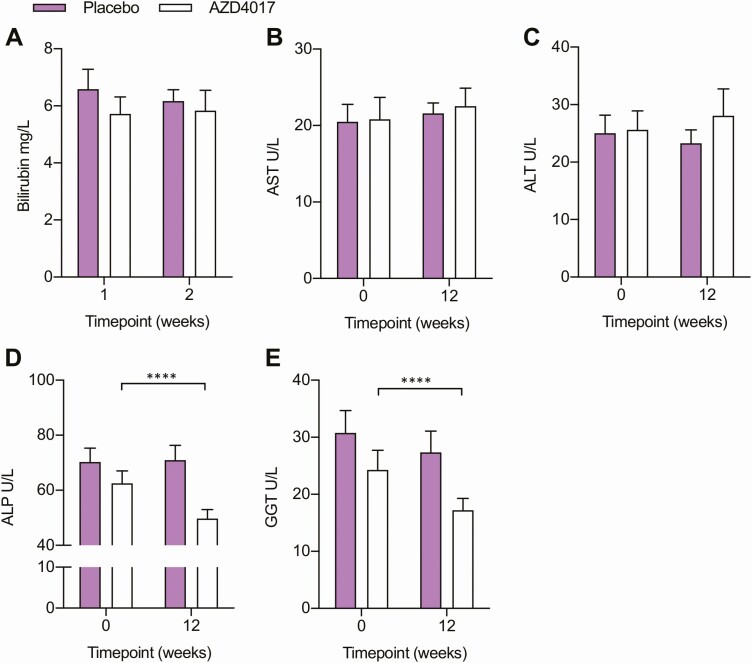
Liver enzymes and kidney function changes following 12 weeks of AZD4017 treatment in IIH patients. Histograms showing (A) bilirubin, (B) AST, (C) ALT, (D) ALP, (E) GGT in IIH patients before and after 12 weeks of either placebo or AZD4017 treatment. ****P < .0001.

Liver and renal function and systemic measures of inflammation were evaluated in patients receiving placebo and AZD4017 throughout the study. In both groups, surrogate markers for liver and renal function were within the normal reference range at baseline. No changes in these markers were apparent in patients receiving placebo at 12 weeks relative to baseline (Fig. 3A-H and (23)). Similarly, no significant changes in bilirubin, ALT and AST levels were apparent in patients receiving AZD4017 at 12 weeks relative to baseline (Fig. 3A-C). However, ALP and GGT significantly decreased in patients receiving AZD4017 relative to baseline (ALP, –20.5%, *P* > .0001; GGT, –29.3%, *P* > .0001) (Fig. 3D and 3E). Within the normal reference range, we observed a significant increase in serum creatinine levels (8.5%, *P* < .0001) and decrease in eGFR (–7.5%, *P* < .001) in patients receiving AZD4017 relative to baseline at 0 weeks, with no correlation observed to lean muscle mass (23). In contrast, no changes were evident in the placebo treated groups relative to baseline.

Serum levels of the inflammatory cytokines IL-6, IL-8, IL-10, MCP-1, and TNF-α were not affected by treatment with AZD4017 (23). Similarly, CSF levels of IL-6, IL-8, and MCP-1 were also unaffected by AZD4017 treatment, with IL-10 and TNF-α levels below the limit of detection. Levels of osteoblast marker osteocalcin and negative regulator of bone formation sclerostin did not change relative to baseline levels in placebo and AZD4017 groups (23). Together, these data indicate that the inhibitor AZD4017 had limited effects on markers of liver function, with reductions in only ALP and GGT, without increasing systemic or CSF inflammation. While not clinically significant, surveillance of renal function using serum creatinine suggests a small but statistically significant decrease in eGFR (within normal parameters) in patients receiving AZD4017.

### Body weight, fat mass, and musculoskeletal parameters

**Figure 4.**
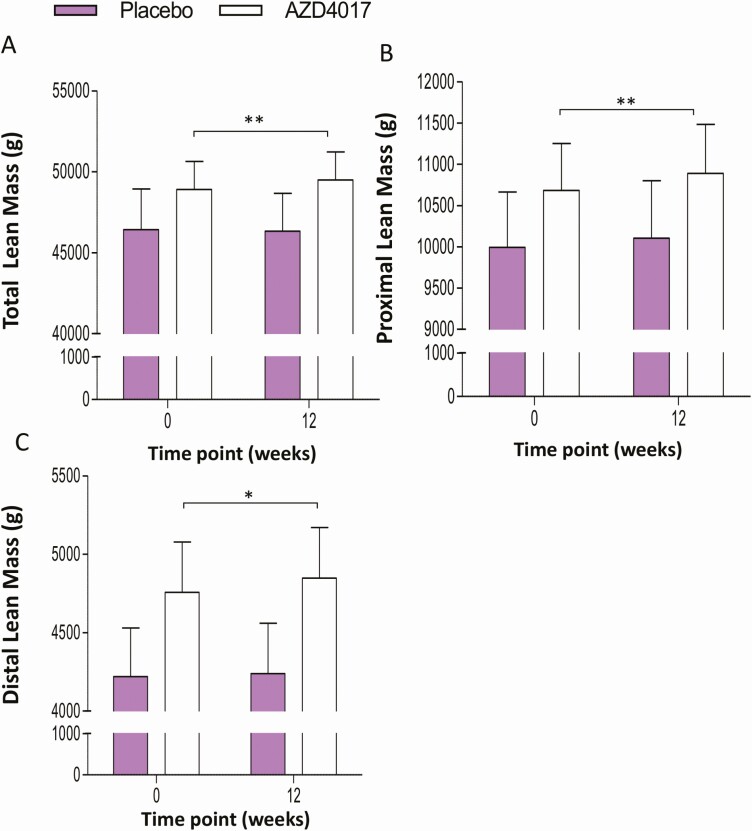
Alterations in (A) total lean mass, (B) proximal lean mass and (c) distal lean mass in grams, determined by DXA following 12 weeks treatments with either placebo (n = 10) or AZD4017 treatment (n = 12) in IIH patients. *P < .05; **P < .01.

To assess the impact of 11β-HSD1 inhibition with AZD4017 on fat and lean mass and mass we examined body composition using DXA measurements. No significant changes were observed for total body weight, BMI, total fat mass, regional fat distribution (trunk, limb, android, or visceral) and bone mineral content in either group relative to baseline (Tables 1 and 2). In contrast, total lean mass increased from baseline to 12 weeks in the treatment group (48 904 ± 6020 g vs 49 493 ± 6008 g, + 1.2%; *P* < .001), but not in the placebo group (Fig. 4A). Closer examination of proximal and distal lean mass revealed that this increase in muscle mass was not specific to either compartment with comparable increases in both groups (proximal, +1.1%; *P* < .05; distal, +1.0%; *P* < .05) (Fig. 4B and 4C). Together, these data indicate that inhibition of 11β-HSD1 with AZD4017 in obese females for 12 weeks did not alter adipose or bone tissue composition but led to a modest increase in muscle mass.

**Table 2.** Body weight and fat mass parameters in an idiopathic intracranial hypertension (IIH) cohort receiving either placebo or the 11β-HSD1 inhibitor AZD4017 at baseline and at 12 weeks

All patients	Placebo (n = 14)/DXA (=10)			AZD4017 (n = 17)/ DXA (n = 12)		
	Baseline	12 weeks	Difference (95% CI)	Baseline	12 weeks	Difference (95% CI)
Weight (kg) ± SD	108.4 ± 42.3	98.7 ± 21.5	0.0 (–1.9 to 2.0), *P* = .9	97.9 ± 21.3	99.1 ± 21.1	1.2 (–0.5 to 2.9), *P* = .2
BMI (weight(kg)/ height(m^2^)) ± SD	41.2 ± 16.6	37.2 ± 8.0	0.1 (–0.5 to 0.7), *P* = .9	37.1 ± 7.1	37.5 ± 6.9	0.4 (–0.3 to 1.1), *P* = .3
Total fat mass (g) ± SD	47 181 ± 15 649.0	46 803.1 ± 16 287.9	–377.9 (–1768.0 to 1012.0), *P* = .7	47 738.9 ± 14 664.2	47 422.9 ± 14 418.1	–316.0 (–1045.0 to 413.0), *P* = .7
Trunk fat mass (g) ± SD	25 699.0 ± 9875.4	25 825.6 ± 10 874.5	126.6 (–925.8 to 1179.0), *P* = .9	24 646.8 ± 7688.3	24 663.9 ± 7561.2	17.2 (–423.1 to 457.4), *P* = .9
Limb fat mass (g) ± SD	20 594.0 ± 6610.0	20 350 ± 6719.0	–243.8 (–863.9 to 376.3), *P* = .5	22 164.0 ± 8054.0	21 823.0 ± 7961.0	–341.1 (–754.4 to 72.2), *P* = .2
Android fat mass (g) ± SD	4605.6 ± 1744.8	4582.5 ± 2177.4	–23.1 (–377.1 to 330.9), *P* = .9	4335.2 ± 1620.4	4436.6 ± 1559.4	101.4 (–79.73 to 282.6), *P* = .6
Gynoid fat mass (g) ± SD	7598.5 ± 2761.4	7607.5 ± 2987.8	9.0 (–298.5 to 316.5), *P* = .9	7848.5 ± 2054.9	7850.5 ± 2157.4	2.0 (–164.3 to 168.3), *P* = .9
Visceral fat mass (g) ± SD	1601 ± 787.7	1596.0 ± 836.3	–4.700 (–90.9 to 81.5), *P* = .8	1268.0 ± 622.0	1306.0 ± 631.5	37.2 (–29.3 to 103.6), *P* = .9
BMC (g) ± SD	2622.1 ± 405.6	2617.0 ± 405.5	–5.1 (–19.4 to 9.2), *P* = .9	2616.2 ± 380.4	2667.5 ± 358.7	51.25 (–63.21 to 165.7), *P* = .3
Total lean mass (kg) ± SD	96.2 ± 21.9	96.1 ± 22.3	–0.2 (–25.1 to 25.5), *P* = .9	98.9 ± 20.3	99.1 ± 20.1	0.3 (–23.3 to 22.8), *P* = .9
Total lean mass proximal (g) ± SD	9992.7 ± 2118.9	10 105.8 ± 2194.9	113.1 (–2564 to 2337), *P* = .9	10 682.7 ± 1885.7	10 890.4 ± 1969.0	207.6 (–2544 to 2129), *P* = .9
Total lean mass distal (g) ± SD	4220.7 ± 977.7	4239.6 ± 1013.6	18.9 (–1261 to 1224), *P* = .9	4757.5 ± 1065.2	4848.3 ± 1069.9	90.8 (–1275 to 1094), *P* = .9

### Steroid metabolism

Inhibition of tissue cortisol activation by 11β-HSD1 may lead to secondary activation of the hypothalamic pituitary adrenal (HPA) axis. While circulating serum cortisol levels appeared unchanged in human studies with 11β-HSD1 inhibitors, there were indications of increased adrenocorticotropin (ACTH) secretion and adrenal androgen production (Table 3) (7, 10, 12). To obtain a comprehensive picture how 11β-HSD1 inhibition may impact on steroid metabolism in humans, we performed serum steroid metabolome profiling by LC-MS/MS in our IIH cohort.

**Table 3.** Serum steroid analysis by LC-MS/MS in an idiopathic intracranial hypertension (IIH) cohort receiving either placebo or the 11β-HSD1 inhibitor AZD4017 at baseline and at 12 weeks

	Serum concentrations in nmol/L (Median ± IQR)					
	Placebo (n = 11–12)			AZD4017 (n = 16)		
	Baseline	12 Weeks	Difference	Baseline	12 Weeks	Difference
Cortisol	185.3 ± 124.7	184.5 ± 105.0	–0.8, *P* = .8	195.6 ± 119.3	158.2 ± 104.1	–37.4, *P* = .6
Cortisone	38.3 ± 12.9	36.9 ± 19.4	–1.4, *P* = .8	43.9 ± 22.8	42.6 ± 27.8	–1.3, *P* = .1
Cortisol:cortisone ratio	4.9 ± 1.4	4.7 ± 1.1	–0.2, *P* = .8	4.8 ± 1.8	3.4 ± 1.6	–1.4, *P* = .01
Cortisol: DHEA ratio	11.0 ± 12.0	9.7 ± 14.0	–1.3, *P* = .1	13.4 ± 8.0	7.0 ± 4.6	–6.4, *P* = .01
Testosterone	0.8 ± 0.9	1.0 ± 1.1	0.2, *P* = .4	0.4 ± 1.0	1.0 ± 1.1	0.6, *P* = .01
A4	3.9 ± 3.6	4.9 ± 4.6	1.0, *P* = .2	3.9 ± 2.9	5.0 ± 2.4	1.1, *P* = .1
DHEA	14.0 ± 15.6	17.6 ± 15.6	3.6, *P* = .5	15.5 ± 9.7	22.7 ± 12.5	7.2, *P* = .08
11OHA4	2.7 ± 2.6	3.8 ± 4.2	1.1, *P* = .9	2.8 ± 3.0	5.7 ± 1.8	2.9, *P* = .01
11KA4	0.9 ± 0.2	0.9 ± 0.4	0.0, *P* = .8	0.9 ± 0.5	1.3 ± 0.4	0.4, *P* = .01

Abbreviations: DHEA, dehydroepiandrosterone; A4, androstenedione; 11OHA4, 11β-hydroxyandrostenedione; 11KA4, 11-ketoandrostenedione.

**Figure 5.**
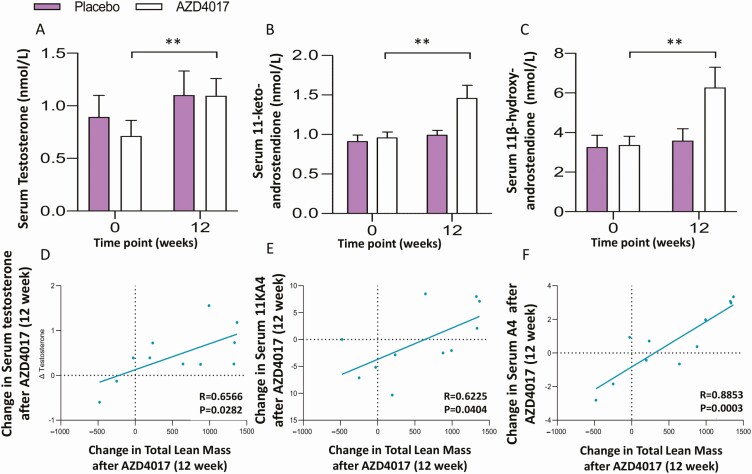
Changes in the androgens (A) testosterone, (B) 11-ketoandrostenedione and (C) 11β-hydroxyandrostenedione in serum following 12 weeks of AZD4017 treatment in IIH patients. Correlations between changes in the androgens (D) testosterone, (E) 11β-hydroxyandrostenedione and (F) androstenedione changes in total lean muscle after treatment with AZD4017 for 12 weeks in IIH patients. Data presented as mean ± SD. **P < .01.

As previously reported, measures of systemic 11β-HSD1 activation of cortisone to cortisol were significantly decreased in line with systemic inhibition of 11β-HSD1 (–1.1; *P* < .001) (Table 3) (6). In addition, we observed a significant decrease in cortisol:DHEA ratio (14.09 ± 0.9 vs 7.8 ± 0.7, *P* < .001) in patients receiving AZD4017 at 12 weeks relative to baseline (Table 3). Analysis of serum androgens revealed an increase following treatment with AZD4017 (Fig. 5A and 5B). This increase did not reach significance for DHEA and androstenedione; however, there was a significant increase in testosterone (0.71 ± 0.15 vs baseline 1.10 ± 0.16 nmol/L, *P* < .01) 11β-hydroxyandrostenedione (3.37 ± 0.44 vs 6.28 ± 1.02 nmol/L, *P* < .01) and 11-ketoandrostenedione (0.96 ± 0.8 vs 1.46 ± 0.17 nmol/L, *P* < .001) (Table 3). The rise in serum androgen levels was accompanied by a trend for higher urinary excretion of androgens and associated metabolites, although differences before and after treatment did not research statistical significance for the AZD4017 subgroup with available data (23). Of note, changes in total lean mass were shown to be positively correlated with the increase in serum testosterone (r = 0.651, *P* = .030), androstenedione (r = 0.855, *P* = .001), and 11-ketoandrostenedione (0.618, *P* = .043) (Fig. 5D-F).

## Discussion

Therapeutic inhibition of 11β-HSD1 activity is of considerable interest in diverse metabolic diseases, for example, obesity, diabetes, metabolic syndrome, and nonalcoholic fatty liver disease (7, 8, 10, 12, 13). The nicotinic amide derived carboxylic acid AZD4017 has been developed as a reversible, highly potent and selective inhibitor of 11β-HSD1 with favorable pharmacokinetics (14). In addition to interest in its application in the management of IIH, it has also recently been evaluated in phase II clinical trials for the management of Iatrogenic Cushing’s syndrome and in postmenopausal women with osteopenia (TICSI, ID: NCT03111810, MICA, ID: MR/K015176/1). In this report, we detail the effects of sustained 11β-HSD1 inhibition with AZD4017 over 12 weeks on parameters of metabolism, body composition, steroid metabolome, and inflammatory cytokine signaling in an overweight patient cohort with IIH.

The use of serial DXA assessments allowed us to analyze changes in body composition with therapeutic 11β-HSD1 inhibition in greater detail than in previous clinical trials. Total lean muscle mass increased in the AZD4017 treatment group over 12 weeks, but no change was observed in the control group. The observed difference of 1.2% lean muscle mass over 12 weeks is considerable, given that the magnitude of change is similar to that observed for muscle wasting in the elderly population of 1% to 2% per year (29). Increased lean muscle mass may result directly from inhibition of glucocorticoid activation within skeletal muscle tissue. Mature skeletal muscle fibers express 11β-HSD1 with cortisol-activating activity as well as glucocorticoid receptors (25). Overall, glucocorticoid stimulation has an antianabolic, atrophic effect on skeletal muscle tissue (30, 31). Our data and previous studies demonstrate that serum cortisol levels are not affected by therapeutic 11β-HSD1 inhibition (7, 10, 12), meaning that circulating cortisol levels cannot adequately account for observed metabolic changes. Instead, alterations in glucocorticoid signaling within muscle tissue may be significant. In support of this hypothesis, skeletal muscle 11β-HSD1 expression has been shown to correlate negatively with muscle size and strength in aging humans (25). To further investigate whether local suppression of 11β-HSD1 activity within skeletal muscle has anticatabolic effects would require examination of tissue-specific pharmacological actions, either in muscle biopsies or through tissue-selective pharmacodynamic studies. While such procedures were outside the remit of our original clinical trial protocol, our results on increased lean muscle mass with AZD4017 treatment strengthen the rationale to address this question in future research.

Alternatively, the increased lean muscle mass observed in the IIH cohort, may result indirectly from systemic changes in androgen concentration and signaling. Here, a rise in circulating androgen levels within the normal range was observed in the AZD4017 treatment group and has been a consistent finding in clinical trials of 11β-HSD1 inhibitors (7, 10, 12). Importantly, androgen receptors are expressed in muscle precursor cells and mature muscle fibers, where they induce myogenesis and muscular hypertrophy and mediate increased lean muscle mass in response to increased circulating androgen levels (32, 33). Supporting this concept, the increases in testosterone and the androgen pro-hormones 11β-hydroxy and 11-ketoandrostenedione metabolites (that are converted to androgenic 11-ketotesosterone) showed a positive correlation with increased lean mass in patients receiving AZD4017. However, we did not measure the circulating concentrations of the active 11-oxygenated androgen 11-ketotestosterone (34), or examine androgen effects in skeletal muscle tissue directly. Acknowledging these limitations, our data indicate that the positive effects of 11β-HSD1 inhibitors on muscle mass may be mediated through increased anabolic androgen signaling, with further research needed to confirm precise pharmacological actions in skeletal muscle tissue. Regardless, these novel observations of increased lean muscle mass observed in patients receiving the 11β-HSD1 inhibitor AZD4017 raise a question of potential benefits in conditions that are associated with muscle wasting. This hypothesis warrants further research.

Previous studies report compensatory activation of the HPA axis as a consequence of 11β-HSD1 inhibition, with modest increases in circulating ACTH and DHEA concentrations (within the respective normal reference ranges) in both male and female patients (7, 10, 12). Elevated ACTH levels were also apparent in this overweight IIH cohort receiving AZD4017 (data reported in (6)). Here, we evaluated serum androgens in this overweight IIH cohort receiving AZD4017. We observed trends towards increased DHEA, with significant increases in testosterone, androstenedione, and 11-oxygenated androgen prohormones, supporting previous findings (7, 10, 12). The accompanying trend for increased urinary excretion of androgens and associated metabolites suggests that reduced renal clearance of androgens does not account for these findings. Although androgen levels remained within the normal range, these data support the concept of a modest HPA axis activation and increased adrenal androgen synthesis. Additional factors that may contribute to our findings include a reduction in the activation of 11-oxygenated androgens by 11β-HSD1 and would require closer investigation in the future (26). In ours, and prior studies, elevated serum androgen concentrations were not associated with clinically evident hirsutism, although the timeframes of this study may be insufficient to adequately assess this, and will merit closer examination in future studies, given prior reports of androgen excess IIH (27).

Modest weight loss or reduction in BMI have been reported in several clinical trials of 11β-HSD1 inhibitors (7, 10, 12, 35, 36). However, we did not observe changes in total body weight or BMI, total body fat mass, or depot–specific fat mass. Similarly, no changes in blood pressure were evident in patients receiving AZD4017 (data not shown). These findings may reflect differences in pharmacodynamic properties of AZD4017 relative to other 11β-HSD1 inhibitors. We observed that systemic administration of AZD4017 had limited capacity to suppress 11β-HSD1 activity in adipose tissue biopsies at 12 weeks (6). This may reflect the reversible nature of this competitive inhibitor, which would allow diffusion of the inhibitor out of the fat biopsy sample into the substantially larger culture medium volume during the ex vivo activity assay, rendering its concentration ineffective. However, AZD4017 effectively blocked 11β-HSD1 activity both in subcutaneous and omental adipose tissue explants at 200 nM, a substantially lower concentration than average serum levels of 4671 nM in the treatment group (6). Alternatively, this could also be consistent with a phenomenon of reduced pharmacological effect with prolonged exposure, which has already been described to limit sustained suppression of 11β-HSD1 activity in human adipose tissue with other systemically administered agents (37). Another consideration could be that this overweight female cohort may be less responsive to weight loss properties of 11β-HSD1 inhibitors compared with patient cohorts with type 2 diabetes and metabolic syndrome.

Bone mineral density and markers of bone turnover were not significantly changed with AZD4017 treatment. The relevance of 11β-HSD1 activity for bone health has become of interest, owing to the well-established detrimental effects of glucocorticoid excess on bone health and animal models suggesting 11β-HSD1 activity inhibits bone formation (30, 38). However, 12 weeks may be an insufficient timeframe in which to measure meaningful changes in bone metabolism. Alternatively, the absence of risk factors for bone mineral loss in this pre-menopausal study cohort may reduce the potential to detect effects of 11β-HSD1 inhibition on bone composition.

Robust improvements in glucose homeostasis have been reported in response to 11β-HSD1 inhibitors in both murine models of diabetes and obesity and in phase II clinical trials in patients with type 2 diabetes and metabolic syndrome, with reductions in fasting insulin, blood glucose, HbA1c, and HOMA2-IR (7, 10, 13, 35, 39-41). No significant changes in any of these parameters were detected in patients receiving placebo or AZD4017 relative to baseline at 12 weeks. As with body weight, these data suggest that as the non-diabetic, healthy obese IIH cohort examined in this trial would be less responsive to this property of AZD4017.

In contrast, we observed favorable changes in lipid profiles in patients receiving AZD4017, closely reflecting those seen in comparable murine models of obesity and clinical trials examining 11β-HSD1 inhibitors (10, 12, 39, 42). Previously, Rosenstock et al. (10) reported a significant decrease in cholesterol, LDL cholesterol, and triglycerides in hyperlipidemic patients, but Shah et al. (12) saw significant decreases in LDL cholesterol and HDL cholesterol, but not triglycerides in patients in higher BMI overweight groups. In this statin naïve overweight IIH cohort, total cholesterol, and LDL cholesterol were reduced relative to baseline in patients receiving AZD4017, whereas HDL cholesterol was increased, supporting the concept that 11β-HSD1 inhibitors may be effective at improving harmful lipid profiles in this context. Consequently, in addition to the direct inhibition of 11β-HSD1 activity and CSF production in the choroid plexus of IIH patients, as proposed by Sinclair et al., secondary improvements in IIH risk factors such as lipid metabolism and obesity may also be realized in patients receiving AZD4017, offering further therapeutic efficacy (15, 18, 19). Particularly, given the increased incidence of adverse cardiovascular outcomes reported in IIH patients relative to matched controls, AZD4017 may prove an effective adjunct in IIH to reduce cardiovascular risk (43).

Lastly, liver, and renal function and measures of systemic and central nervous system inflammation were assessed in this overweight IIH cohort receiving AZD4017. These revealed some positive effects on markers of liver function, with significant decreases in both ALP and GGT. The mechanism underpinning this remains unclear, although previous studies in mice have demonstrated protection from fat accumulation and hepatic steatosis within the livers of mice with transgenic deletion of 11β-HSD1 (44). However, in this study, the reported changes were modest and their clinical relevance remains unclear. Similarly, changes in serum creatinine, and hence eGFR, were also small and not clinically significant in this population with normal renal function at baseline. It is important to note that serum creatinine levels are significantly influenced by lean muscle mass as well as renal clearance, reducing their accuracy as a marker of kidney function in patients with extremes of BMI or changing lean body mass, as seen in our cohort (45). On further scrutiny, observed serum creatinine changes were not concordant with changes in lean muscle mass or changes in serum urea (data reported in (6)) to support either a non-renal or renal explanation respectively. No changes in renal markers have been reported in previous studies examining 11β-HSD1 inhibitors. Hence, our data can neither confirm nor deny that inhibition of 11β-HSD1 with AZD4017 may negatively impact on kidney function. Further research will be needed to address this important question and should involve more specific measures of glomerular filtration rate.

11β-HSD1 activity, which is potently stimulated by inflammatory stimuli, is emerging as an important feedback regulator of local tissue damage related to inflammation (46-48). To address concerns that 11β-HSD1 inhibition may precipitate an adverse proinflammatory phenotype, we examined a wider array of inflammatory markers than previous clinical trials with 11β-HSD1 inhibitors. No changes related to AZD4017 treatment over 12 weeks were detected for the inflammatory cytokine levels systemically or in the central nervous system. Hence, in a cohort without significant chronic inflammatory activity at baseline, our study offers further evidence that therapeutic 11β-HSD1 inhibition does not precipitate inflammation.

### Conclusions

In summary, this study provides a metabolic profile for the 11β-HSD1 inhibitor AZD4017 in an overweight IIH cohort in an extended phase II clinical trial. In particular, we demonstrate that it reduces harmful lipid profiles while increasing androgen levels and lean muscle mass, without having adverse systemic inflammatory or hepatic actions although a clinically minimal decline in renal function was observed. These beneficial metabolic changes and increases in lean muscle mass, not only represent a reduction in risk factors associated with IIH, but also indicate new potential applications for this class of 11β-HSD1 inhibitor in the management of sarcopenia and age related muscle wasting.

## Data Availability

Restrictions apply to the availability of some or all data generated or analyzed during this study to preserve patient confidentiality or because they were used under license. The corresponding author will on request detail the restrictions and any conditions under which access to some data may be provided.

## Abbreviations

11β-HSD1: 11β-hydroxysteroid dehydrogenase type 1
ACTH: adrenocorticotropin
ALP: alkaline phosphatase
ALT: alanine aminotransferase
AST: aspartate transaminase
BMI: body mass index
CK: creatinine kinase
CSF: cerebrospinal fluid
CV: coefficient of variation
DHEA: dehydroepiandrosterone
DXA: dual-energy X-ray absorptiometry
eGFR: estimated glomerular filtration rate
GGT: gamma-glutamyl transferase
HbA1c: glycated hemoglobin
HDL: high-density lipoprotein
HOMA2-IR: homeostasis model assessment of insulin resistance
HPA: hypothalamic pituitary adrenal
IC50: median inhibitory concentration
IIH: idiopathic intracranial hypertension
IL: interleukin
LC-MS/MS: liquid chromatography-tandem mass spectrometry
MTBE: tert-butyl methyl ether
OC: osteocalcin
SC: sclerostin
TNF: tumor necrosis factor
UPLC: ultrahigh-performance liquid chromatography

## Additional Information

***Disclosure Summary:*** A.J.S. reports advisory board fees from Novartis and Allergan related to migraine. Speaker fees on migraine for Novartis. Invex therapeutics, company director with salary and stock options (2019, 2020). S.M.P. reports—Invex therapeutics advisory board (2019); Heidelberg engineering speaker fees (2019). O.G. reports Consultancy work for Invex therapeutics (2020). R.J.F. works for AstraZeneca. Other authors report no conflicts of interest in the subject matter to report. The views expressed are those of the authors and not necessarily those of the NIHR or the Department of Health and Social Care UK. The funders of the study had no role in the: design and conduct of the study; collection, management, analysis, and interpretation of the data; preparation, review, or approval of the manuscript; decision to submit the manuscript for publication. The views expressed are those of the authors and not necessarily those of the NIHR or the Department of Health and Social Care.

***Clinical Trial Information:*** NCT02017444 (registered December 20, 2013).
